# The Present Position of the Territorial Medical Service

**Published:** 1910-05-28

**Authors:** 


### The Royal Army Medical Corps Section.

### THE PRESENT POSITION OF THE TERRITORIAL MEDICAL SERVICE.

As the retirement of Sir Alfred Keogh from the position of Director-General of the Army Medical Service practically took place at December 31, 1909, it is a fitting occasion to refer to the progress that has been made in the formation of that Citizen Military Medical Service that was inaugurated by him, and must always be associated with his name. It is not necessary to refer in detail to the circumstances that afforded him the opportunity of establishing a modern and up-to-date medical service that would embody and represent the present-day views upon what is one of the most important accessories of every army. As is well known, the occasion was furnished by the disappearance of the old Volunteer force and the substitution for it of an army for home defence raised on a Territorial system.

In the execution of his task Sir Alfred Keogh laid down certain principles which had been lost sight of in the old Volunteer medical system, but were very properly regarded by him as fundamental. In the first place, the new Citizen Medical Service must be organised on exactly similar lines to that of the Royal Army Medical Corps. Secondly, owing to the fact that many of the duties the members of the force would be called upon to perform in time of war are those daily undertaken by them in the active practice of civil life, there should be no need to call upon them to undergo special training in time of peace. Lastly, while providing for co-ordination of the work of the several branches of the profession and ensuring harmonious action, care was to be taken that the duties allocated to members of the medical service in time of war should be those they were accustomed to carry out in their everyday professional work.

With these principles as the ground work of his plan, Sir Alfred Keogh proceeded to construct the organisation of his new Citizen Medical Service. Following the Royal Army Medical Corps as a pattern, he provided for the following details: (1) A new medical and sanitary service with combatant units; (2) field medical units, consisting of field ambulances and cavalry ambulances for service with infantry divisions and cavalry or mounted brigades; (3) sanitary companies; and (4) general hospitals. If, however, the Royal Army Medical Corps pattern had been closely followed the Territorial Medical Service would have had its clearing hospitals in addition to the above-mentioned units; but these were intentionally omitted, so as to give the Red Cross organisation of the country an opportunity of coming in and furnishing the necessary assistance for the removal of the sick and wounded from the field ambulances to the general hospitals in the rear. This the Red Cross Society is now busily engaged in arranging for by means of voluntary aid detachments, as recently described in The Hospital. As soon as his scheme was sufficiently worked out, Sir Alfred Keogh took the very proper and diplomatic course of laying it before the profession as a whole, and soliciting their friendly criticism on it, indicating that he was open to receive any suggestions for improving it. Some minor modifications were made in it, but taken generally it was very favourably received and cordially approved of by the whole body of civilian practitioners, and they have given it a most loyal and patriotic support. Reference to the Army List clearly indicates this, for it shows nearly all the battalions with their medical establishments and the different divisions and mounted brigades with their required field ambulances under divisional and mounted brigade commanders. Sanitary companies, too, have also been formed for the sanitation of camps of concentration and similar camps under local commanders, while to all the divisions administrative medical officers, with staff medical officers and sanitary officer, have been appointed to co-ordinate and supervise the work of all the units belonging to their divisions. In connection with none of these units has greater enthusiasm been shown than in the case of the general hospitals. Twenty-three in number, they have all been organised under administrative officers of the Territorial Medical Corps with staffs of physicians and surgeons from the large civil hospitals in the locality, with a nursing staff from a Territorial Force Nursing Service, and with other subordinate *personnel* from the Territorial Force and Voluntary Aid Societies. Such a result is in every way creditable to the medical and nursing professions, and indicates that Sir Alfred Keogh's scheme has met with very general approval. As already pointed out, there are gaps in it that the Red Cross Society is busily engaged in filling up; but with the completion of the voluntary aid detachments and a proper arrangement for the supply of convalescent homes, we feel justified in saying that the Territorial Force would be adequately provided with medical aid and succour should invasion take place and war occur by this scheme, if carried out properly and in its entirety.

Upon the duties of the Territorial Medical Service we need not dwell, as they have been detailed from time to time in the pages of The Hospital; but there is one point that we would emphasise and consider should be kept steadily in view, and that is the essential difference between the new Territorial Army and all the various forms of Volunteer Forces that have preceded it. This is supposed to consist in the real preparedness of the former for warfare. From the first the military authorities have announced that the Territorial Army is intended, in the event of invasion, to be in a position at once to take the field fully equipped as a fighting force composed of the right proportions of the various arms and furnished with all the other equally essential portions of an army on active service. Of these latter none are more important than a thoroughly effective Territorial Medical Corps, for if invasion has to be repelled it must be by fighting, and this means wounds, while service in the field must be accompanied by sickness. There are no greater hindrances to military activity and efficiency than these two factors of sick and wounded, so that it is an absolute necessity to have them removed as quickly as possible. This can only be attained by the prompt and skilled help of a properly organised and efficient medical service well trained in the means for preserving the health of the troops, in the care and treatment of the sick and wounded, in the replenishing of medical and surgical equipment, and in the collection of the sick and wounded in the fighting area and their removal from the scene of active operations. We need not point out that such prompt and skilled assistance can only be furnished by a *personnel* who have had ample opportunities given them for training and for making themselves efficient, and that to allow of this all units should be furnished with suitable drill-hall accommodation and with ample and up-to-date equipment; but we feel justified in saying that the Territorial Medical Service has still grounds of complaint on both of these points, and that in both these essentials there is still much to be desired. Again, in the case of the officers too great a demand should not be made on their time in the matter of clerical work. To have to furnish numerous, and often unnecessary, returns, stands in the way of their time being devoted, as it ought to be, to the subject of field work and the best methods of utilising the general medical resources in an area of operations. Especially does this apply to administrative medical officers of divisions, several of whom have not yet been given staff medical officers, with the result that a good deal of their energy and time has to be devoted to office work instead of studying what has been very properly termed the "strategical and tactical employment of the medical service." We feel that these points must be pressed upon the notice of the military authorities to ensure their doing what is their proper duty to a service whose members have shown a commendable spirit of patriotism, and have brought to their work great enthusiasm and spirit. It would be a matter of great regret if Sir Alfred Keogh's splendid work should fail through want of official support, and that the Territorial Medical Service should not continue to be a lasting monument of the tact and high professional abilities of one of the ablest Medical Director-Generals the British Army has ever had.

